# Consumption of antibiotics in the community, European Union/European Economic Area, 1997–2017

**DOI:** 10.1093/jac/dkab172

**Published:** 2021-08-01

**Authors:** Robin Bruyndonckx, Niels Adriaenssens, Ann Versporten, Niel Hens, Dominique L Monnet, Geert Molenberghs, Herman Goossens, Klaus Weist, Samuel Coenen, the ESAC-Net study group, Reinhild Strauss, Eline Vandael, Stefana Sabtcheva, Arjana Tambić Andrašević, Isavella Kyriakidou, Jiří Vlček, Ute Wolff Sönksen, Elviira Linask, Emmi Sarvikivi, Karima Hider-Mlynarz, Doreen Richter, Flora Kontopidou, Mária Matuz, Gudrun Aspelund, Karen Burns, Filomena Fortinguerra, Elīna Dimina, Jolanta Kuklytė, Marcel Bruch, Peter Zarb, Stephanie Natsch, Hege Salvesen Blix, Anna Olczak-Pieńkowska, Ana Silva, Gabriel Adrian Popescu, Tomáš Tesař, Milan Čižman, Antonio López Navas, Vendela Bergfeldt, Susan Hopkins

**Affiliations:** 1 Laboratory of Medical Microbiology, Vaccine & Infectious Disease Institute (VAXINFECTIO), University of Antwerp, Antwerp, Belgium; 2 Interuniversity Institute for Biostatistics and statistical Bioinformatics (I-BIOSTAT), Data Science Institute, Hasselt University, Hasselt, Belgium; 3 Centre for General Practice, Department of Family Medicine & Population Health (FAMPOP), University of Antwerp, Antwerp, Belgium; 4 Centre for Health Economic Research and Modelling Infectious Diseases, Vaccine & Infectious Disease Institute (VAXINFECTIO), University of Antwerp, Antwerp, Belgium; 5 Disease Programmes Unit, European Centre for Disease Prevention and Control, Stockholm, Sweden; 6 Interuniversity Institute for Biostatistics and statistical Bioinformatics (I-BIOSTAT), Catholic University of Leuven, Leuven, Belgium

## Abstract

**Objectives:**

Data on antibiotic consumption in the community were collected from 30 EU/EEA countries over two decades. This article reviews temporal trends, seasonal variation, presence of change-points and changes in the composition of the main antibiotic groups.

**Methods:**

For the period 1997–2017, data on consumption of antibiotics, i.e. antibacterials for systemic use (ATC group J01), in the community, aggregated at the level of the active substance, were collected using the WHO ATC/DDD methodology (ATC/DDD index 2019). Consumption was expressed in DDD per 1000 inhabitants per day and in packages per 1000 inhabitants per day. Antibiotic consumption was analysed based on ATC-3 groups, and presented as trends, seasonal variation, presence of change-points and compositional changes.

**Results:**

In 2017, antibiotic consumption in the community expressed in DDD per 1000 inhabitants per day varied by a factor 3.6 between countries with the highest (Greece) and the lowest (the Netherlands) consumption. Antibiotic consumption in the EU/EEA did not change significantly over time. Antibiotic consumption showed a significant seasonal variation, which decreased over time. The number of DDD per package significantly increased over time. The proportional consumption of sulphonamides and trimethoprim (J01E) relative to other groups significantly decreased over time, while the proportional consumption of other antibacterials (J01X) relative to other groups significantly increased over time.

**Conclusions:**

Overall, antibiotic consumption in the community in the EU/EEA did not change during 1997–2017, while seasonal variation consistently decreased over time. The number of DDD per package increased during 1997–2017.

## Introduction

Over time, misuse of antibiotics has led to antimicrobial resistance, resulting in treatment failure, increased costs of care and elevated mortality. In order to fight this global problem, comparable and reliable information of antibiotic consumption is essential.^1^ This article presents data from the European Surveillance of Antimicrobial Consumption Network (ESAC-Net^2^, formerly ESAC) on antibiotic consumption in the community (i.e. primary care sector) for 30 EU/EEA countries in 1997–2017. It updates previous ESAC studies published in 2006 and 2011.^3^^,^^4^ The objective of this study was to analyse temporal trends, seasonal variation and the presence of change-points in antibiotic consumption in the community for the period 1997–2017, as well as to analyse the composition of antibiotic consumption over time.

## Methods

The methods for collecting and analysing the data are described in the introductory article of this series.^5^ In summary, data on consumption of antibiotics, i.e. antibacterials for systemic use (ATC group J01) aggregated at the level of the active substance, were collected using the WHO ATC/DDD methodology (ATC/DDD index 2019^6^) and expressed in DDD per 1000 inhabitants per day. In addition, where data were available, antibiotic consumption was also expressed in packages per 1000 inhabitants per day.

Antibiotics were classified in 10 ATC groups: β-lactam antibacterials, penicillins (J01C), other β-lactam antibacterials (J01D), macrolides, lincosamides and streptogramins (J01F), quinolone antibacterials (J01M), tetracyclines (J01A), sulphonamides and trimethoprim (J01E), other antibacterials (J01X), amphenicols (J01B), aminoglycoside antibacterials (J01G) and combinations of antibacterials (J01R). Due to limited consumption of the last three groups, these were combined and their consumption was presented as ‘other antibiotics’.

The evolution of the number of DDD per package over time was assessed using a linear mixed model. The temporal trend, seasonal variation and presence of change-points in antibiotic consumption were assessed using a non-linear change-point mixed model fitted to quarterly data expressed in DDD per 1000 inhabitants per day from 1997 to 2017.^7^ The relative proportions of the main groups were assessed through a compositional data analysis modelling yearly data expressed in DDD per 1000 inhabitants per day from 1997 to 2017.^8^

## Results

An overview of consumption of antibacterials for systemic use (ATC J01) in the community, expressed in DDD and packages per 1000 inhabitants per day for all participating countries between 1997 and 2017 is available as Supplementary data at *JAC* Online (Tables S1 and S2, respectively).

### Antibiotic consumption in the community in 2017

**Figure 1.**
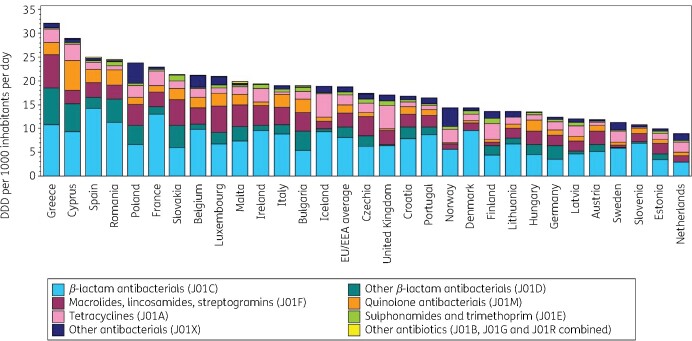
Consumption of antibiotics (ATC J01) in the community, expressed in DDD (ATC/DDD index 2019) per 1000 inhabitants per day, 30 EU/EEA countries, 2017. For Czechia, 2015 data are used. For Slovakia, 2016 data are used. For Cyprus and Romania, total care data, i.e. community and hospital sector combined, are used. For Ireland, nitrofurantoin (J01XE01) consumption is not included. For Slovenia, sulphonamide and trimethoprim (J01E) consumption is not included.

The proportion of antibiotics (ATC J01) represented by the main ATC-3 groups is presented in Table 1. Figure 1 shows the consumption of antibiotics in the community, expressed in DDD per 1000 inhabitants per day, for 30 EU/EEA countries in 2017. Antibiotic consumption varied by a factor of 3.6 between countries with the highest (32.15 DDD per 1000 inhabitants per day in Greece) and the lowest (8.94 DDD per 1000 inhabitants per day in the Netherlands) consumption in 2017.

**Table 1.** Distribution of antibiotic consumption (ATC J01) in the community by ATC level-3, EU/EEA, 2017

Group (ATC code)	Proportion (%)
Tetracyclines (J01A)	11.28
Amphenicols (J01B)	0.03
β-Lactam antibacterials, penicillins (J01C)	42.30
Other β-lactam antibacterials (J01D)	11.62
Sulphonamides and trimethoprim (J01E)	2.85
Macrolides, lincosamides and streptogramins (J01F)	16.10
Aminoglycoside antibacterials (J01G)	0.17
Quinolone antibacterials (J01M)	9.47
Combinations of antibacterials (J01R)	0.11
Other antibacterials (J01X)	6.07

The most frequently consumed antibiotics in the community in 2017 were the β-lactam antibacterials, penicillins (J01C), with proportional consumption (out of total consumption) ranging from 27.88% (Poland) to 66.39% (Denmark). The proportional consumption ranged from 0.22% in Denmark to 23.96% in Greece for other β-lactam antibacterials (J01D), from 4.77% in Sweden to 26.29% in Luxembourg for macrolides, lincosamides and streptogramins (J01F), from 2.46% in Norway to 21.49% in Cyprus (total care data, i.e. community and hospital sector combined) for quinolone antibacterials (J01M), from 2.51% in Italy to 28.22% in the United Kingdom for tetracyclines (J01A), from 0.07% in Lithuania to 7.55% in Finland for sulphonamides and trimethoprim (J01E), from 0.17% in Ireland (nitrofurantoin (J01XE01) consumption not included) to 27.46% in Norway for other antibacterials (J01X), and from 0.01% in Portugal to 1.51% in Malta for other antibiotics (J01B, J01G and J01R combined).

**Figure 2.**
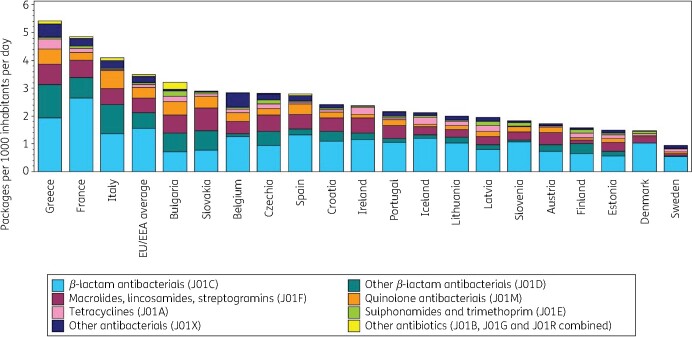
Consumption of antibiotics (ATC J01) in the community, expressed in packages per 1000 inhabitants per day, 20 EU/EEA countries, 2017. For Czechia, 2015 data are used. For Slovakia, 2016 data are used. For Ireland, nitrofurantoin (J01XE01) consumption is not included.

Figure 2 shows consumption of antibiotics (J01) in the community expressed in packages per 1000 inhabitants per day for 20 EU/EEA countries in 2017. Based on packages rather than DDD, Greece also showed the highest consumption (5.42 packages per 1000 inhabitants per day) while Sweden showed the lowest consumption (0.95 packages per 1000 inhabitants per day). The ranking of countries for their consumption expressed in DDD per 1000 inhabitants per day or in packages per 1000 inhabitants per day was similar (Table 2). The lowest mean number of DDD per package was observed for France and Italy (both 4.7 DDD per package) and the highest for Sweden (11.8 DDD per package). In the EU/EEA, the number of DDD per package significantly increased over time during 1997–2017, with the steepness of this increase significantly reducing over the time period.

**Table 2.** Ranking of consumption of antibiotics (ATC J01) in the community, expressed in DDD or packages per 1000 inhabitants per day, 20 EU/EEA countries, 2017^a^

Country	Greece	France	Italy	Bulgaria	Slovakia	Belgium	Czechia	Spain	Croatia	Ireland	Portugal	Iceland	Lithuania	Latvia	Slovenia	Austria	Finland	Estonia	Denmark	Sweden
Ranking by packages per 1000 inhabitants per day	1	2	3	4	5	6	7	8	9	10	11	12	13	14	15	16	17	18	19	20
Ranking by DDD per 1000 inhabitants per day	1	3	7	8	4	5	10	2	11	6	12	9	15	16	19	17	14	20	13	18
Number of DDD per package	5.9	4.7	4.7	5.9	7.3	7.4	6.2	8.9	6.9	8.1	7.6	8.9	6.8	6.2	5.9	6.9	8.6	6.7	9.7	11.8

a For Czechia, 2015 data are used. For Slovakia, 2016 data are used. For Cyprus and Romania, total care data are used. For Ireland, nitrofurantoin (J01XE01) consumption was not included.

### Longitudinal data analysis, 1997–2017

**Figure 3.**
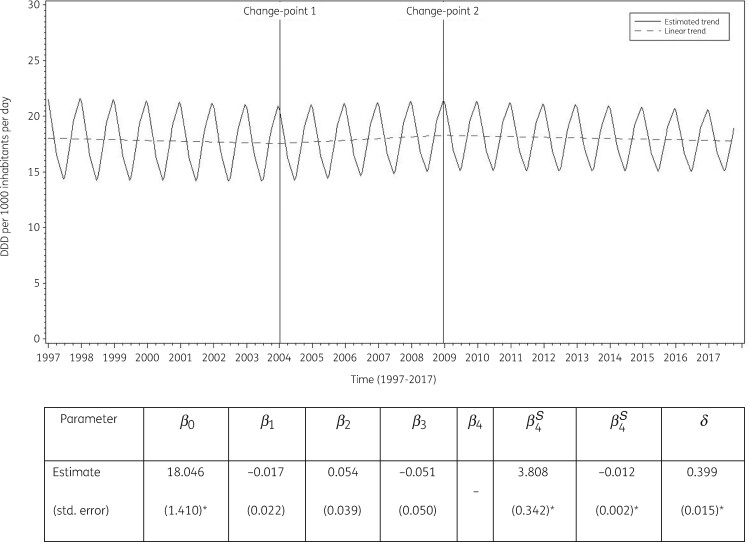
Estimated trend (solid line) and linear trend (dashed line) of consumption of antibiotics (ATC J01) in the community based on quarterly data, 25 EU/EEA countries, 1997–2017. β_0_, predicted consumption in the first quarter of 1997; β_1_, predicted increase (if positive)/decrease (if negative) in consumption per quarter; β_2_, predicted difference in slope after versus before the first change-point; β_3_, predicted difference in slope after versus before the second change-point; β_4_, predicted difference in slope after versus before the third change-point; β_0_^S^, predicted amplitude of the upward winter and downward summer peak in consumption; β_1_^S^, predicted increase (if positive)/decrease (if negative) of the amplitude of the upward winter and downward summer peak in consumption per quarter; δ, shift in timing of the upward winter and downward summer peak from one year to another. *Statistically significant at significance level 0.05.

The best fit was obtained for a model including two change-points: one in the first quarter of 2004 and another in the last quarter of 2008. The final model fits the observed data well (Figure S1). The longitudinal data analysis estimated an average antibiotic consumption (ATC J01) in the EU/EEA of 18.046 (SE 1.410) DDD per 1000 inhabitants per day in 1997, which did not change significantly over time: −0.017 (SE 0.022) DDD per 1000 inhabitants per day per quarter between 1997 and the first quarter of 2004; +0.037 (SE 0.041) DDD per 1000 inhabitants per day per quarter between the second quarter of 2004 and the last quarter of 2008; and −0.014 (SE 0.059) DDD per 1000 inhabitants per day per quarter afterwards. In addition, the analysis showed significant seasonal variation with an amplitude of 3.808 (SE 0.342) DDD per 1000 inhabitants per day, which decreased significantly over time: −0.012 (SE 0.002) DDD per 1000 inhabitants per day per quarter (Figure 3).

Based on the final fitted model, antibiotic consumption in the community in 1997 was significantly above average in Belgium, Greece and Slovakia, and significantly below average in Austria, Denmark, Estonia, Germany, the Netherlands, Sweden and the United Kingdom (observed profiles shown in Figures S2 and S3). The seasonal variation was significantly larger than average in Belgium, Greece, Hungary, Italy, Luxembourg and Slovakia, and significantly smaller than average in Denmark, Estonia, Finland, Iceland, Ireland, the Netherlands, Slovenia, Sweden and the United Kingdom. The decrease in antibiotic consumption between 1997 and the first quarter of 2004 was significantly larger than average in Belgium and Spain. The increase in antibiotic consumption between the second quarter of 2004 and the last quarter of 2008 was significantly larger than average in Belgium, Denmark and Lithuania. There were no countries showing a significantly larger than average decrease from the first quarter of 2009 onwards.

### Compositional data analysis, 1997–2017

The proportional consumption of sulphonamides and trimethoprim (J01E) significantly decreased over time relative to consumption of all other groups (Table 3). The proportional consumption of other antibacterials (J01X) significantly increased over time relative to that of all other groups, except for the other antibiotics (J01B, J01G and J01R combined). In addition, the proportional consumption of β-lactam antibacterials (J01C) significantly increased over time relative to that of tetracyclines (J01A) and significantly decreased over time relative to that of quinolone antibacterials (J01M). The proportional consumption of tetracyclines (J01A) significantly decreased over time relative to that of macrolides, lincosamides and streptogramins (J01F), quinolone antibacterials (J01M) and other antibiotics (J01B, J01G and J01R combined). The proportional consumption of other β-lactam antibacterials (J01D) significantly decreased relative that of quinolone antibacterials (J01M). The proportional consumption of macrolides, lincosamides and streptogramins (J01F) significantly decreased relative to that of quinolone antibacterials (J01M).

**Table 3.** Change in the composition of the consumption of antibiotics (ATC J01) in the community, expressed in DDD per 1000 inhabitants per day (ATC/DDD index 2019), 30 EU/EEA countries, as a function of time during the period 1997–2017

	J01A	J01C	J01D	J01E	J01F	J01M	J01X	Other
J01A		**−0.023**	−0.013	**0.031**	**−0.021**	**−0.036**	**−0.072**	**−0.039**
J01C	**0.023**		0.010	**0.054**	0.003	**−0.013**	**−0.049**	−0.015
J01D	0.013	−0.010		**0.044**	−0.007	**−0.023**	**−0.054**	−0.025
J01E	**−0.031**	**−0.054**	**−0.044**		**−0.051**	**−0.067**	**−0.103**	**−0.069**
J01F	**0.021**	−0.003	0.007	**0.051**		**−0.016**	**−0.051**	−0.018
J01M	**0.036**	**0.013**	**0.023**	**0.067**	**0.016**		**−0.036**	−0.002
J01X	**0.072**	**0.049**	**0.054**	**0.103**	**0.051**	**0.036**		0.033
Other	**0.039**	0.015	0.025	**0.069**	0.018	0.002	−0.033	

Values are estimated changes in the log ratio of the row versus column group of antibiotics with increasing time. Bold type indicates a statistically significant effect; positive values represent an increase and negative values represent a decrease.

J01A, tetracyclines; J01C, β-lactam antibacterials; penicillins J01D, other β-lactam antibacterials; J01E, sulphonamides and trimethoprim; J01F, macrolides, lincosamides and streptogramins; J01M, quinolone antibacterials; J01X, other antibacterials; Other, amphenicols (J01B), aminoglycoside antibacterials (J01G) and combinations of antibacterials (J01R), all combined.

Trends of proportional consumption of antibiotic groups in individual countries are shown in Figure S4. When comparing the composition of the consumption of antibiotics (ATC J01) in 2017 with that in 2009, the proportion of sulphonamides and trimethoprim (J01E) decreased in most of the participating EU/EEA countries, with the largest decreases observed for Slovenia (−8.92%), Latvia (−5.32%) and Iceland (−5.17%). However, increases were also observed, with the largest increases reported for Slovakia (+3.54%; 2016 data), Romania (+1.94%; total care data, i.e. community and hospital sector combined) and Italy (+1.49%). The proportion of other antibacterials (J01X) increased in most countries, with the largest increases observed for Poland (+11.00%) and Norway (+8.29%). Decreases were also observed, with the largest decreases reported for Lithuania (−8.24%) and Finland (−1.51%). The proportion of β-lactam antibacterials (J01C) increased in most countries, with the largest increases observed for France (+11.91%), Portugal (+10.14%) and Croatia (+9.34%). Decreases were also observed, with the largest decreases observed for Poland (−7.84%), Bulgaria (−7.78%) and Luxembourg (−5.72%). The proportions of tetracyclines (J01A), quinolone antibacterials (J01M), macrolides, lincosamides and streptogramins (J01F) and other β-lactam antibacterials (J01D) increased for some countries while they decreased for others. More detailed results for the main antibiotic groups are presented in separate articles in this series.^9–13^

## Discussion

We demonstrated that EU/EEA antibiotic consumption in the community expressed in DDD per 1000 inhabitants per day did not change significantly during 1997–2017. In an international study analysing the trend of total (i.e. covering both community and hospital sector) antibiotic consumption in 76 countries expressed in DDD per 1000 inhabitants per day, consumption remained stable among high-income countries between 2000 and 2015. Given that the majority of the EU/EEA countries belong to the high-income category, this is consistent with the results of our study.^14^

The analyses also revealed seasonality of antibiotic consumption in the community, which was observed for all EU/EEA countries and decreased significantly over time. This may result in a reduction in the incidence of *Clostridioides* (*Clostridium*) *difficile* infections as predicted by mathematical modelling.^15^ While a limited amount of seasonal variation could be associated with seasonality in bacterial pathogens, the extent of the observed seasonality suggests inappropriate prescribing for viral (mostly respiratory) infections during the winter season.^16^^,^^17^ In addition, conclusions should not be drawn based on a single quality indicator. In a separate article in this series, we evaluated 13 quality indicators, thus allowing for more-solid conclusions.^18^

We explored countries’ ranking based on antibiotic consumption expressed in DDD per 1000 inhabitants per day or in packages per 1000 inhabitants per day, and found these to be similar. However, since the average number of DDD per package significantly increased between 1997 and 2017, this finding is time-dependent. Both metrics can be used to summarize a country’s overall antibiotic consumption. In countries solely dispensing complete packages, a package may be considered as a surrogate for a prescription (and hence one treated patient), although some patients may only need half a package while others may need two or more packages. However, in countries where pharmacies dispense the exact number of singe units to fulfil a prescription, e.g. the Netherlands and the UK, the number of packages is not a suitable metric and the number of prescriptions instead of the number of packages should be monitored. The DDD is a technical unit defined as the assumed average maintenance dose per day for the main indication of a substance in adults. For antibiotics, the DDD is based on the treatment of infections of moderate severity. Its standardization and updates to take into account changes in prescribing practices make the DDD an international standard for surveillance of consumption of medicinal products, including antibiotics.^19^ Nevertheless, the DDD is not a suitable metric for children since the DDD are defined for adults, and specific indicators have been defined for antibiotic consumption in children.^20^ Because our aim in this series was to provide an in-depth overview—in composition and over time—of antibiotic consumption, we used the most complete data, i.e. data expressed in DDD per 1000 inhabitants per day, for further analyses.

The inter-country variability in consumption of antibiotics (ATC J01) expressed in DDD per 1000 inhabitants per day was substantial. This has also been reported for European countries that are not part of the ESAC-Net, but covered by the WHO Europe Antimicrobial Medicines Consumption Network in which consumption varied by a factor of 4.3, from 36.4 DDD per 1000 inhabitants per day in Turkey to 8.5 in Azerbaijan.^21^

Despite changes in DDD values in 2019, which mostly affected the β-lactam antibacterials, penicillins (J01C), this group remained the most consumed antibiotic group in all 30 EU/EEA countries (Czechia 2015 data, Slovakia 2016 data). This finding confirms that of previously published studies.^1^^,^^2^ While the proportional consumption of sulphonamides and trimethoprim (J01E) decreased in most countries, the proportional consumption of other antibacterials (J01X) increased in most countries. Given that total antibiotic consumption did not change significantly over time between 1997 and 2017, this implies that consumption of antibiotics from one subgroup was merely replaced by consumption of antibiotics from another subgroup, rather than being reduced overall. More detailed discussions on the main antibiotic groups are given in separate articles in this series.^9–13^

To further broaden our understanding of appropriate prescribing and the variations between and within EU/EEA countries over time, more detailed data on antibiotic use (e.g. linked to patient records containing age, gender, indication for prescribing and comorbidities) and antimicrobial resistance rates, and information on treatment guidelines at the national level would be needed.

Nevertheless, the data on antibiotic consumption in the community in EU/EEA countries, available from ESAC-Net, together with the tools of compositional and longitudinal data analyses provided in this series may enable countries to evaluate their own antibiotic consumption by linking their country-specific change-points to possible explanations, e.g. targeted surveillance initiatives or the implementation of national guidelines. In addition, it allows countries to evaluate the impact of public awareness campaigns and important policy changes for a more prudent use of antibiotics in the community.^22^

While this study has a major strength in the number of participating countries and the completeness of their recorded data, it has a major limitation in the aggregated nature of the collected data. Because the data only contained information on countries’ antibiotic consumption rates, we were unable to distil any findings by age (e.g. children or elderly) or gender. In addition, the aggregation prevents a distinction between high-but-appropriate and inappropriate antibiotic prescribing, which is deemed quintessential in fighting overconsumption of antibiotics. For a discussion on the limitations of the statistical approach used in this study and potential explanations for the common change-points detected through these analyses, we refer to the tutorial paper in this series.^7^

In conclusion, while antibiotic consumption did not change significantly during 1997–2017, seasonal variation decreased.

## Supplementary Material

dkab172_Supplementary_Data
