# Supplementary material for: Consumption of antibiotics in the community, European Union/European Economic Area, 1997–2017
Source: J Antimicrob Chemother. 2021 Aug 1;76(Suppl 2):ii7–ii13. doi: 10.1093/jac/dkab172 (PMC8314117; doi:10.1093/jac/dkab172)
Supplement: dkab172_Supplementary_Data [file dkab172_supplementary_data.docx]

**Supplementary data**

**Table S1. Consumption of antibacterials for systemic use (ATC J01) in the community, expressed in DDD (ATC/DDD index 2019) per 1000 inhabitants per day, 30 EU/EEA countries, 1997-2017.**

| **Country** | **1997** | **1998** | **1999** | **2000** | **2001** | **2002** | **2003** | **2004** | **2005** | **2006** | **2007** | **2008** | **2009** | **2010** | **2011** | **2012** | **2013** | **2014** | **2015** | **2016** | **2017** |
| --- | --- | --- | --- | --- | --- | --- | --- | --- | --- | --- | --- | --- | --- | --- | --- | --- | --- | --- | --- | --- | --- |
| Austria | - | 11.70 | 12.15 | 11.35 | 10.77 | 10.66 | 11.18 | 11.24 | 12.83 | 12.59 | 12.98 | 13.29 | 13.63 | 13.13 | 12.67 | 12.19 | 14.17 | 12.09 | 12.06 | 11.42 | 11.92 |
| Belgium | 22.42 | 23.24 | 23.01 | 22.24 | 20.73 | 20.73 | 20.49 | 19.38 | 20.43 | 20.11 | 20.96 | 22.68 | 22.16 | 23.11 | 23.60 | 23.94 | 22.57 | 22.36 | 22.75 | 22.53 | 21.14 |
| Bulgaria | - | - | *15.11* | *20.24* | *22.66* | *17.29* | *15.54* | *16.44* | *15.55* | 15.29 | 16.73 | 17.49 | 15.91 | 15.84 | 16.96 | 16.11 | 17.30 | 18.60 | 18.82 | 17.64 | 18.94 |
| Croatia | - | - | - | - | 16.46 | 19.93 | 20.51 | 20.29 | 21.13 | 19.36 | 20.46 | 20.77 | 19.39 | 17.18 | 16.52 | 18.26 | 17.61 | 17.77 | 18.03 | 17.07 | 16.83 |
| Cyprus | - | - | - | - | - | - | - | - | - | *27.14* | *28.65* | *28.69* | *29.16* | *26.26* | *26.86* | *25.09* | *23.95* | *22.19* | *26.59* | *28.37* | *28.88* |
| Czechia | - | 16.68 | 16.89 | - | - | - | 15.20 | 14.30 | 15.55 | 14.46 | 14.87 | 15.87 | 16.62 | 16.01 | 16.51 | 15.66 | 16.90 | 17.12 | 17.37 | - | - |
| Denmark | 11.79 | 12.32 | 11.75 | 11.95 | 12.42 | 12.84 | 13.12 | 13.67 | 14.18 | 14.72 | 15.35 | 15.07 | 15.02 | 15.87 | 16.69 | 15.70 | 15.66 | 15.17 | 15.31 | 15.16 | 14.33 |
| Estonia | - | - | - | - | *14.37* | 10.35 | 9.76 | 9.21 | 10.39 | 10.02 | 10.69 | 10.46 | 9.72 | 9.81 | 10.75 | 10.35 | 10.33 | 10.22 | 10.50 | 10.38 | 9.94 |
| Finland | 18.40 | 17.56 | 17.56 | 18.10 | 18.63 | 16.90 | 17.59 | 16.28 | 16.97 | 16.36 | 17.12 | 16.48 | 16.56 | 17.01 | 18.55 | 17.98 | 16.93 | 16.59 | 15.76 | 15.03 | 13.59 |
| France | 27.93 | 28.27 | 28.79 | 28.37 | 28.12 | 27.13 | 24.50 | 23.05 | 24.31 | 23.23 | 23.84 | 23.33 | 24.40 | 23.21 | 23.35 | 24.01 | 24.13 | 23.12 | 23.79 | 23.87 | 22.97 |
| Germany | 12.26 | 12.41 | 12.69 | 12.74 | 11.86 | 11.77 | 12.90 | 12.12 | 13.54 | 12.57 | 13.33 | 13.45 | 13.78 | 13.39 | 13.09 | 13.70 | 14.49 | 13.37 | 13.11 | 12.84 | 12.34 |
| Greece | 22.23 | 22.49 | 25.33 | 26.27 | 26.40 | 27.51 | 28.00 | *29.81* | *31.14* | *36.89* | *38.89* | *40.39* | 34.60 | *35.56* | 31.63 | 28.20 | 27.99 | 29.17 | 31.26 | 30.96 | 32.15 |
| Hungary | - | 16.55 | 21.11 | 16.62 | 16.48 | 15.05 | 16.79 | 16.01 | 17.11 | 15.05 | 13.41 | 13.42 | 13.86 | 13.62 | 13.80 | 13.00 | 13.37 | 14.02 | 14.70 | 13.33 | 13.43 |
| Iceland | *22.19* | *23.14* | *21.74* | *20.52* | *20.00* | *20.64* | *18.74* | *19.39* | *20.89* | 17.80 | 17.07 | 18.45 | 17.16 | *19.82* | *19.81* | *19.67* | *19.44* | 17.11 | 17.59 | 18.17 | 18.84 |
| Ireland^a^ | - | 14.48 | 15.70 | 15.34 | 16.19 | 16.23 | 17.41 | 17.63 | 17.74 | 18.35 | 19.70 | 19.31 | 17.79 | 17.39 | 19.21 | 19.46 | 20.05 | 19.51 | 21.29 | 20.38 | 19.35 |
| Italy | - | - | 21.38 | 20.95 | 22.09 | 20.92 | 21.80 | 21.05 | 22.10 | 22.31 | 22.87 | 23.59 | 23.73 | 23.02 | 23.10 | 22.51 | 23.32 | 22.63 | 22.38 | 21.84 | 19.02 |
| Latvia | - | - | - | - | - | 9.67 | - | 10.43 | 10.74 | 9.75 | 10.49 | 9.69 | 9.33 | 9.95 | 10.78 | 10.96 | 11.34 | 10.62 | 11.14 | 11.06 | 12.05 |
| Lithuania | - | - | - | - | - | - | - | - | - | *22.09* | *19.72* | *21.00* | *16.20* | *14.41* | *15.48* | 13.27 | 15.11 | 13.13 | 13.64 | 13.51 | 13.56 |
| Luxembourg | 24.30 | 25.43 | 25.01 | 24.12 | 24.31 | 23.98 | 24.70 | 21.47 | 22.55 | 21.29 | 23.07 | 22.99 | 23.75 | 23.19 | 23.33 | 23.14 | 23.14 | 21.60 | 21.85 | 21.36 | 20.93 |
| Malta | - | - | - | - | - | - | - | - | - | - | 15.02 | 17.89 | 18.60 | 18.10 | 20.08 | 19.50 | 20.67 | 20.45 | 18.76 | 18.39 | 19.79 |
| Netherlands | 9.05 | 8.90 | 8.98 | 8.82 | 8.83 | 8.76 | 8.72 | 8.74 | 9.37 | 9.68 | 9.86 | 10.01 | 10.11 | 10.01 | 10.13 | 10.09 | 9.61 | 9.42 | 9.49 | 9.25 | 8.94 |
| Norway | - | 15.04 | - | - | 15.29 | 15.42 | 15.30 | 15.40 | 16.41 | 14.51 | 15.16 | 15.21 | 14.89 | 15.41 | 16.11 | 16.53 | 15.83 | 15.54 | 15.43 | 14.89 | 14.37 |
| Poland | - | 18.72 | 19.85 | 19.88 | 21.57 | 18.28 | - | 16.87 | 17.19 | - | 18.71 | 17.41 | 20.08 | 18.00 | 18.22 | 19.88 | 20.50 | 19.88 | 22.81 | 20.73 | 23.79 |
| Portugal | 20.00 | 20.12 | 21.65 | 21.25 | 20.89 | 22.43 | 21.40 | 20.36 | 20.68 | 19.10 | - | 18.99 | 19.13 | 18.61 | 19.23 | 18.71 | 16.10 | 16.60 | 17.34 | 17.54 | 16.37 |
| Romania^b^ | - | - | - | - | - | - | - | - | - | - | - | - | *9.65* | - | *26.48* | *25.94* | *26.76* | *26.62* | *28.05* | *24.42* | *24.50* |
| Slovakia | - | - | 22.54 | 24.61 | 25.76 | 23.78 | 24.50 | 20.03 | 22.31 | 19.94 | 22.07 | 20.74 | 21.26 | - | *21.37* | 17.86 | 21.13 | 18.88 | 21.98 | 21.30 | - |
| Slovenia | 15.22 | 16.60 | 16.80 | 15.46 | 14.86 | 13.98 | 14.55 | 14.36 | 13.82 | 12.46 | 13.45 | 12.62 | 11.93 | 11.81 | 11.88 | 11.78 | 11.91 | 11.62 | 11.86 | 10.65 | 10.74 |
| Spain^c^ | 17.70 | 17.11 | 16.55 | 15.76 | 14.93 | 14.87 | 15.51 | 15.09 | 15.47 | 14.96 | 15.94 | 15.77 | 15.67 | 16.19 | 16.60 | 15.66 | 16.16 | 17.07 | 17.49 | 25.63 | 25.01 |
| Sweden | 14.30 | 15.16 | 15.44 | 15.21 | 15.47 | 14.88 | 14.31 | 14.18 | 14.48 | 14.95 | 15.07 | 14.22 | 13.69 | 13.76 | 13.89 | 13.71 | 12.65 | 12.48 | 11.92 | 11.67 | 11.26 |
| United Kingdom | 14.93 | 14.23 | 13.10 | 12.68 | 13.06 | 13.07 | 13.34 | 13.26 | 13.60 | 13.52 | 14.47 | 14.91 | 15.20 | 16.46 | 16.50 | 17.66 | 18.31 | 18.54 | 17.90 | 17.45 | 17.06 |

**-**, no consumption reported; Numbers reported in *italic* are total care data, i.e. community and hospital sector combined; ^a^Data for Ireland do not include nitrofurantoin (J01XE01) consumption; ^b^Data for Romania have a coverage in 2009 limited to 30-40%; ^c^Data for Spain include private prescriptions from 2016 onwards.

**Table S2. Consumption of antibacterials for systemic use (ATC J01) in the community, expressed in packages per 1000 inhabitants per day, 23 EU/EEA countries, 2006-2017.**

| **Country** | **2006** | **2007** | **2008** | **2009** | **2010** | **2011** | **2012** | **2013** | **2014** | **2015** | **2016** | **2017** |
| --- | --- | --- | --- | --- | --- | --- | --- | --- | --- | --- | --- | --- |
| Austria | - | 1.89 | 1.90 | 1.97 | 1.89 | 1.82 | 1.76 | 2.04 | 1.74 | 1.73 | 1.65 | 1.73 |
| Belgium^a^ | - | 2.39 | 2.53 | 2.53 | 2.55 | 2.56 | 2.58 | 2.47 | 2.41 | 2.38 | 3.13 | 2.84 |
| Bulgaria | 3.58 | 3.78 | 3.75 | 3.26 | 3.29 | 3.36 | 3.12 | 3.26 | 3.44 | 3.38 | 3.24 | 3.22 |
| Croatia | - | 3.43 | 3.34 | 3.04 | 2.59 | 2.48 | 2.67 | 2.61 | 2.66 | 2.65 | 2.46 | 2.42 |
| Czechia | - | 2.08 | - | - | 2.92 | 2.96 | 2.66 | 2.68 | 2.81 | 2.82 | - | - |
| Denmark | - | 1.81 | 1.76 | 1.72 | 1.79 | 1.86 | 1.70 | 1.68 | 1.62 | 1.59 | 1.55 | 1.47 |
| Estonia | 2.00 | 2.06 | 1.86 | 1.70 | 1.74 | 1.84 | 1.78 | 1.75 | 1.69 | 1.69 | 1.62 | 1.49 |
| Finland | - | - | 1.88 | 1.90 | 1.97 | 2.13 | 2.04 | 1.92 | 1.90 | 1.79 | 1.73 | 1.58 |
| France | - | - | - | - | 5.22 | 5.26 | 5.27 | 5.21 | 4.94 | 5.10 | 5.05 | 4.86 |
| Greece | *8.66* | *9.27* | *9.33* | 5.30 | *7.80* | 4.87 | 4.27 | 4.30 | 4.82 | 4.93 | 5.12 | 5.42 |
| Iceland | - | - | - | - | *2.63* | *2.66* | *2.78* | *2.65* | 2.06 | 2.09 | 2.13 | 2.11 |
| Ireland^b^ | - | 2.68 | - | - | 2.37 | 2.54 | 2.58 | 2.59 | 2.39 | 2.55 | 2.57 | 2.37 |
| Italy | - | - | 5.52 | - | 5.30 | 5.17 | 5.01 | 5.15 | 4.95 | 4.87 | 4.67 | 4.09 |
| Latvia | - | - | - | - | 1.82 | 1.84 | 1.80 | 1.86 | 1.74 | 1.79 | 1.78 | 1.95 |
| Lithuania | - | *3.47* | *3.91* | *2.93* | *2.87* | *3.04* | 2.11 | 2.32 | 2.01 | 2.04 | 2.05 | 2.01 |
| Luxembourg | 2.95 | 3.02 | 2.94 | 2.98 | 2.86 | 2.78 | 2.72 | 2.71 | 2.56 | 2.52 | 2.57 | - |
| Netherlands^c^ | - | - | 1.51 | 1.53 | - | - | - | - | - | - | - | - |
| Portugal | - | - | 2.60 | 2.55 | 2.50 | 2.51 | 2.46 | 2.10 | 2.15 | 2.24 | 2.25 | 2.15 |
| Slovakia | - | - | - | - | - | *3.54* | 2.63 | 3.10 | 2.91 | 3.09 | 2.90 | - |
| Slovenia | - | 2.39 | 2.20 | 2.12 | 2.06 | 2.02 | 1.96 | 1.97 | 1.91 | 1.94 | 1.85 | 1.83 |
| Spain^d^ | - | - | - | - | 2.21 | 2.24 | 2.07 | 2.05 | 1.98 | 1.99 | 2.89 | 2.79 |
| Sweden | - | - | - | 1.19 | 1.19 | 1.18 | 1.15 | 1.05 | 1.01 | 0.99 | 0.98 | 0.95 |
| United Kingdom | - | - | - | - | - | - | - | - | - | 0.11 | - | - |

**-**, no consumption reported; Numbers reported in *italic* are total care data, community and hospital sector combined; ^a^Data for Belgium are slightly overestimated from 2016 onwards (nursing homes counting units versus packages before 2016); ^b^Data for Ireland do not include nitrofurantoin (J01XE01) consumption; ^c^Data for the Netherlands are based on average package size; ^d^Data for Spain include private prescriptions from 2016 onwards.

**
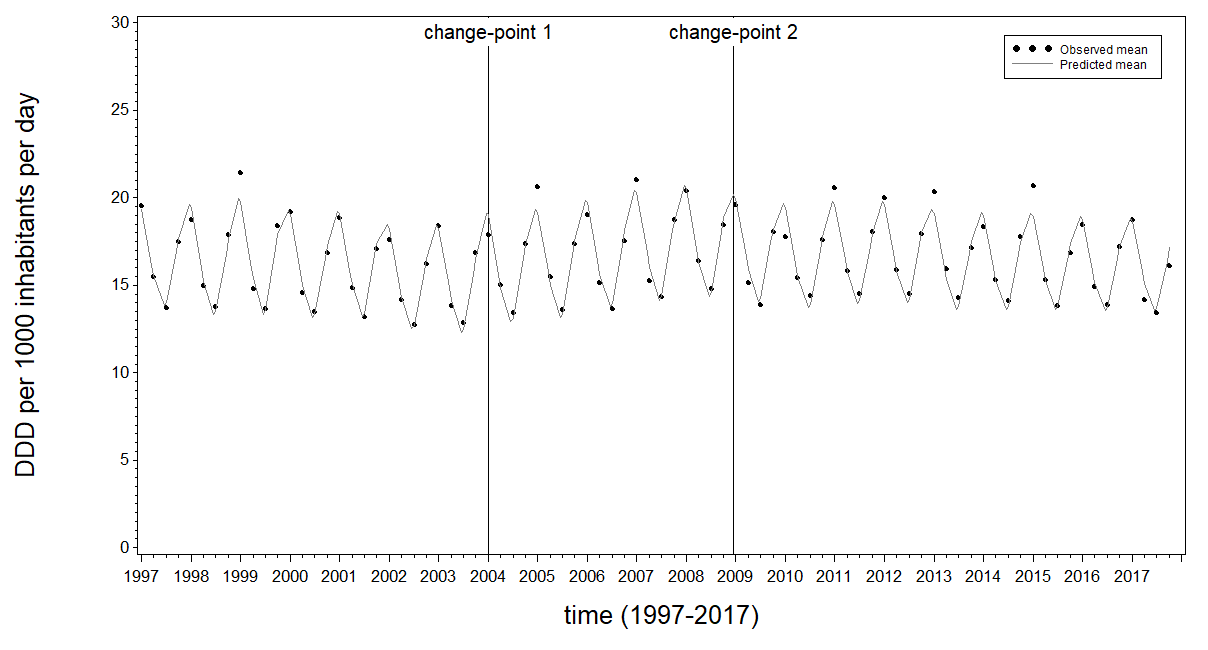
**

**Figure S1. Average of observed (dots) and predicted (solid line) consumption of antibacterials for systemic use (ATC J01) in the community expressed in DDD (ATC/DDD index 2019) per 1000 inhabitants per day and based on quarterly data, 25 EU/EEA countries, 1997-2017.**

**
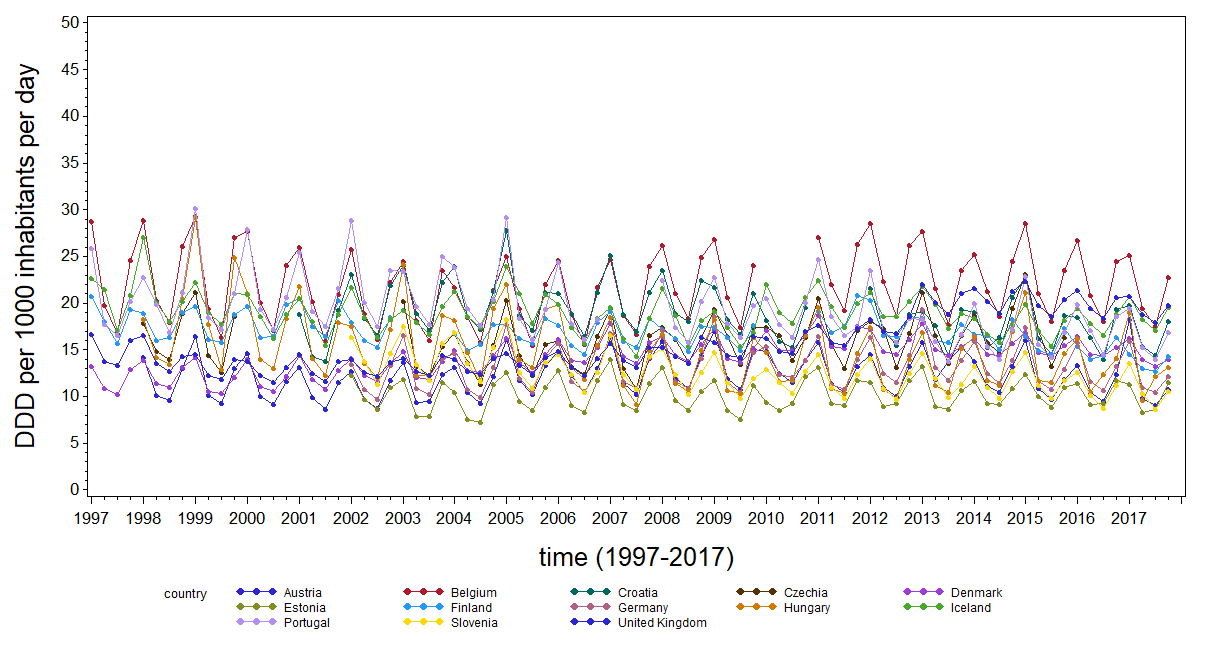
**

**Figure S2. Seasonal variation in consumption of antibacterials for systemic use (ATC J01) in the community, expressed in DDD (ATC/DDD index 2019) per 1000 inhabitants per day, 13 EU/EEA countries reporting consumption per quarter for at least 15 years, 1997-2017.**


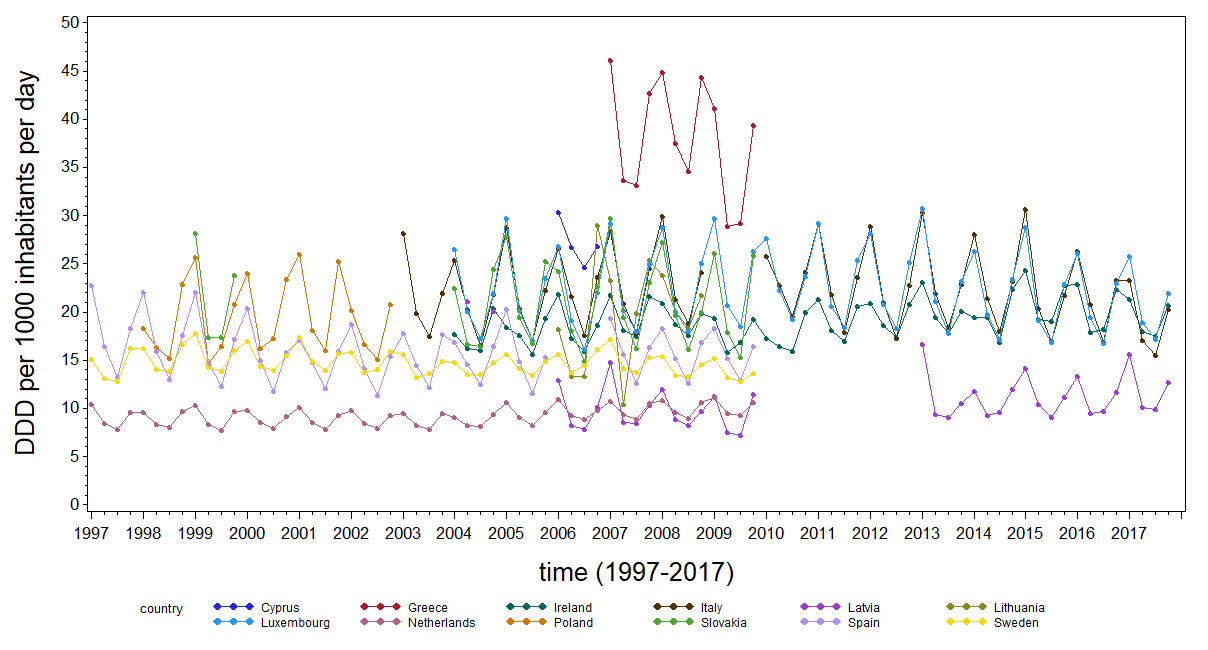


**Figure S3. Seasonal variation in consumption of antibacterials for systemic use (ATC J01) in the community, expressed in DDD (ATC/DDD index 2019) per 1000 inhabitants per day, 12 EU/EEA countries reporting consumption per quarter for less than 15 years, 1997*-*2017. For Cyprus, total care data, i.e. community and hospital sector combined, are used. For Ireland, nitrofurantoin (J01XE01) consumption is not included. For Spain, private prescriptions are included from 2016 onwards.**

^
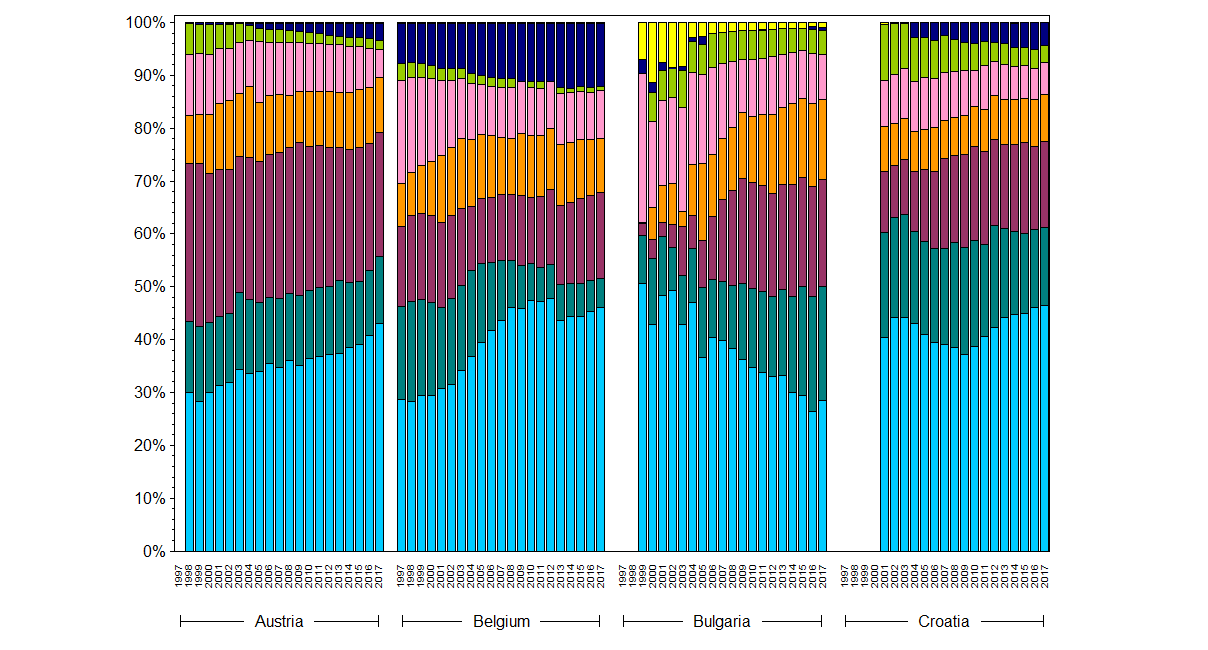
^

^
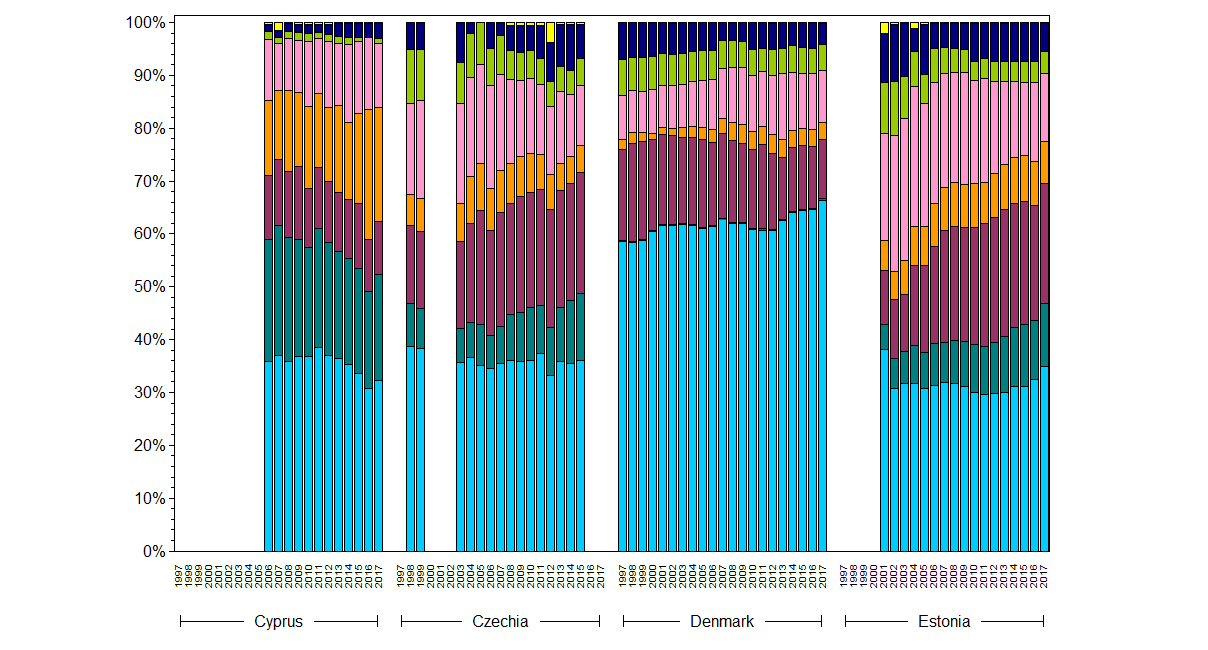
^

= β-lactam antibacterials, penicillins (J01C), = other β-lactam antibacterials (J01D),
 = macrolides, lincosamides and streptogramins (J01F), = quinolone antibacterials (J01M), = tetracyclines (J01A),
 = sulfonamides and trimethoprim (J01E), = other antibacterials (J01X), = other antibiotics (concatenation of amphenicols (J01B), aminoglycoside antibacterials (J01G) and combinations of antibacterials (J01R)).

**Figure S4. Composition of antibacterials for systemic use (ATC J01) consumption in the community, expressed in DDD (ATC/DDD index 2019) per 1000 inhabitants per day, 30 EU/EEA countries, 1997-2017. For Cyprus and Romania, total care data, i.e. community and hospital sector combined, are used. For Ireland, nitrofurantoin (J01XE01) consumption is not included. For Spain, private prescription are included from 2016 onwards. For Romania, data have a coverage in 2009 limited to 30-40%.**

^
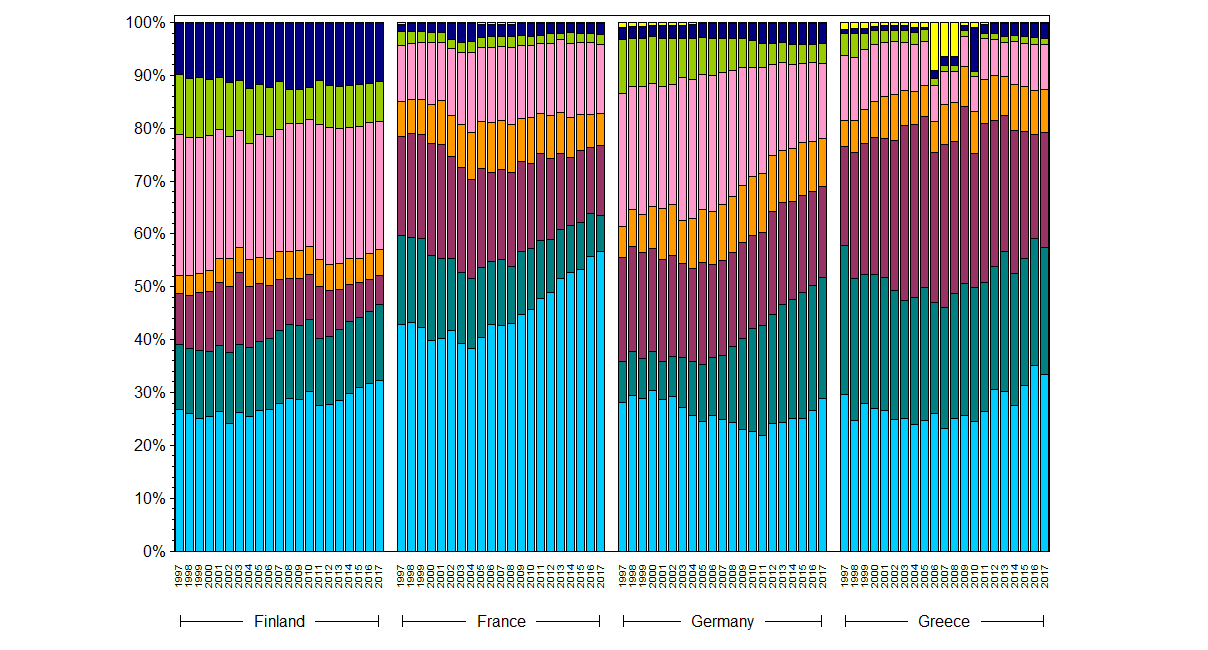

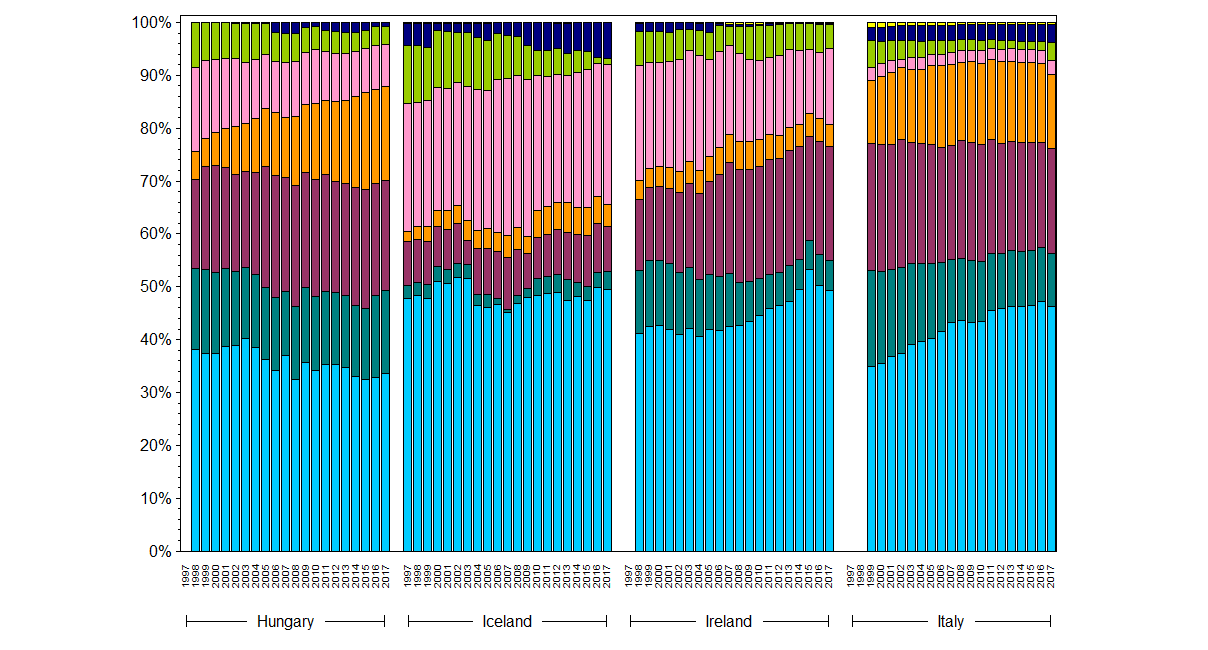
^

= β-lactam antibacterials, penicillins (J01C), = other β-lactam antibacterials (J01D),
 = macrolides, lincosamides and streptogramins (J01F), = quinolone antibacterials (J01M), = tetracyclines (J01A),
 = sulfonamides and trimethoprim (J01E), = other antibacterials (J01X), = other antibiotics (concatenation of amphenicols (J01B), aminoglycoside antibacterials (J01G) and combinations of antibacterials (J01R)).

**Figure S4.** Continued

^
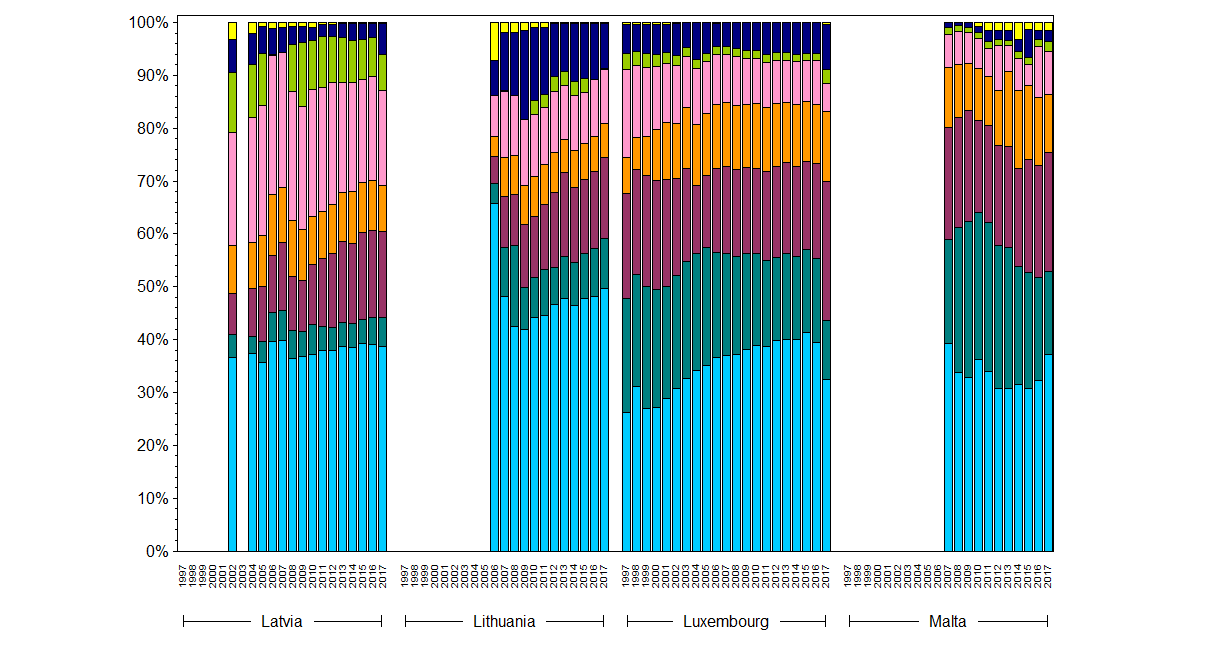

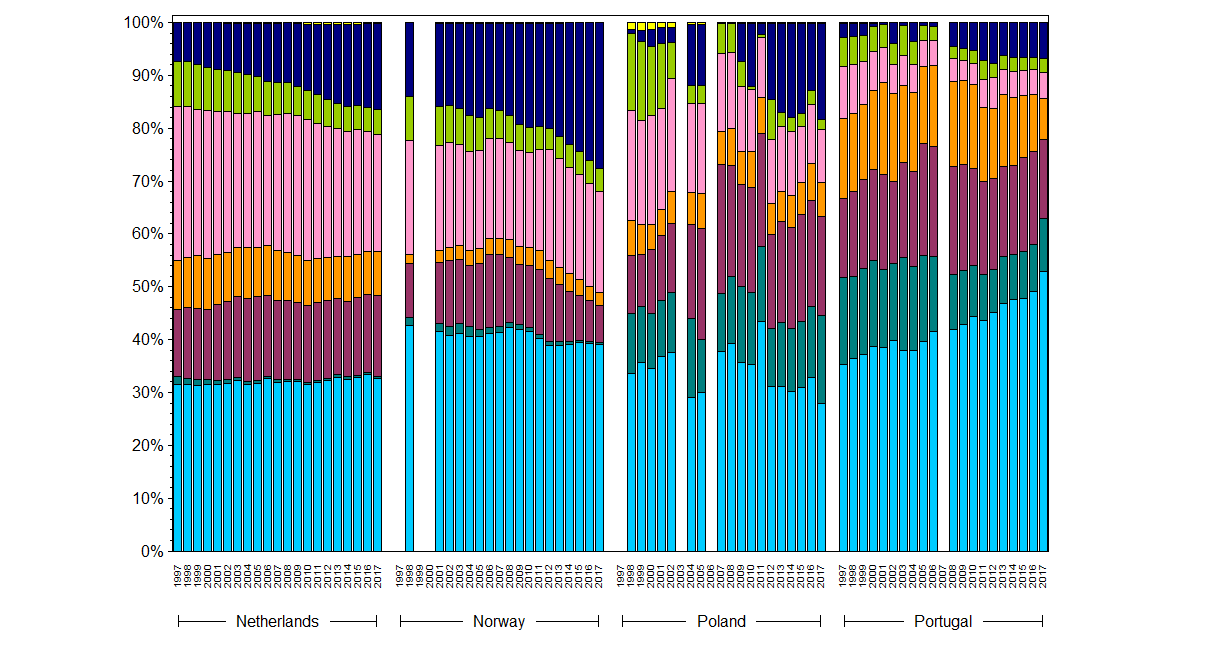
^

= β-lactam antibacterials, penicillins (J01C), = other β-lactam antibacterials (J01D),
 = macrolides, lincosamides and streptogramins (J01F), = quinolone antibacterials (J01M), = tetracyclines (J01A),
 = sulfonamides and trimethoprim (J01E), = other antibacterials (J01X), = other antibiotics (concatenation of amphenicols (J01B), aminoglycoside antibacterials (J01G) and combinations of antibacterials (J01R)).

**Figure S4.** Continued

^
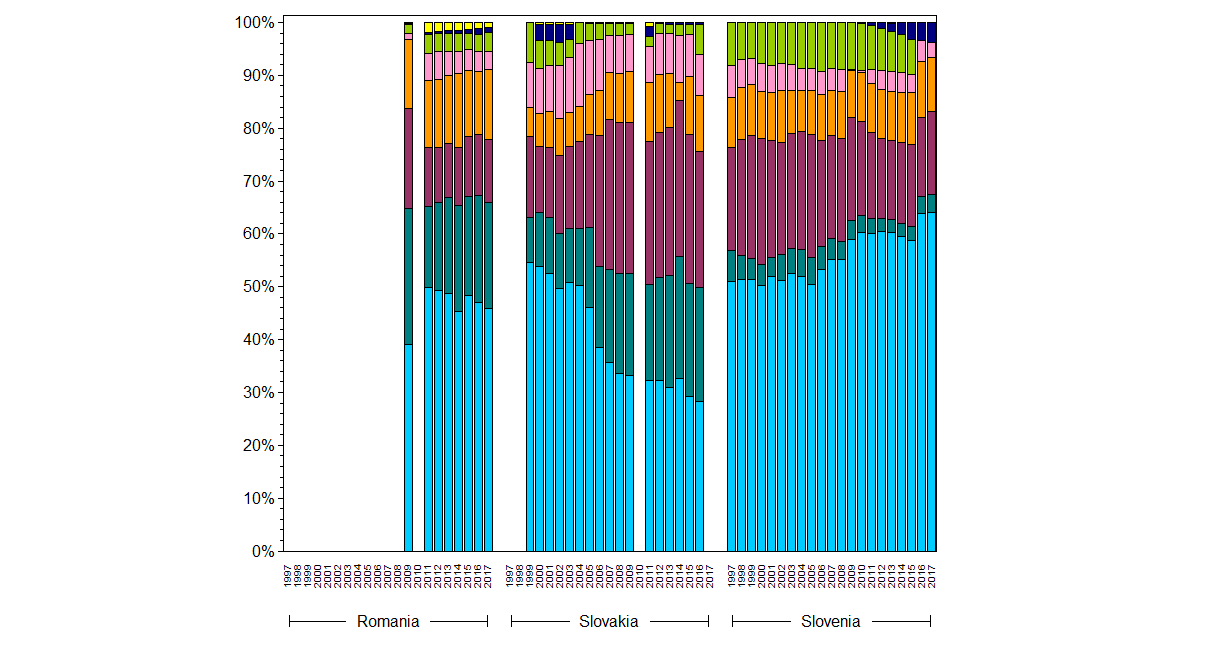
^


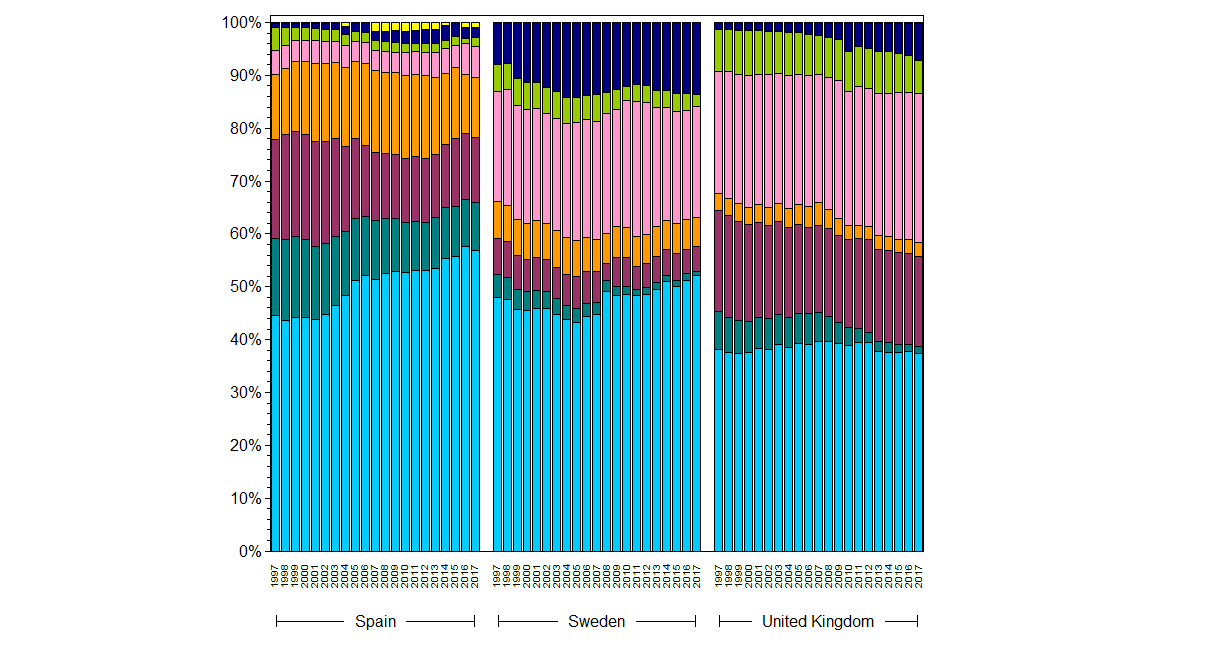


= β-lactam antibacterials, penicillins (J01C), = other β-lactam antibacterials (J01D),
 = macrolides, lincosamides and streptogramins (J01F), = quinolone antibacterials (J01M), = tetracyclines (J01A),
 = sulfonamides and trimethoprim (J01E), = other antibacterials (J01X), = other antibiotics (concatenation of amphenicols (J01B), aminoglycoside antibacterials (J01G) and combinations of antibacterials (J01R)).

**Figure S4.** Continued
